# Supplementary material for: A fault-intrusion-tolerant system and deadline-aware algorithm for scheduling scientific workflow in the cloud
Source: PeerJ Comput Sci. 2021 Nov 2;7:e747. doi: 10.7717/peerj-cs.747 (PMC8576554; doi:10.7717/peerj-cs.747)
Supplement: Supplemental Information 6 [file peerj-cs-07-747-s006.zip › Myproject1.1/docs/org/cloudbus/cloudsim/network/datacenter/NetworkVm.html]

NetworkVm


JavaScript is disabled on your browser.


- Overview
- Package
- Class
- Use
- Tree
- Deprecated
- Index
- Help

- Prev Class
- Next Class

- Frames
- No Frames

- All Classes

- Summary:
- Nested |
- Field |
- Constr |
- Method

- Detail:
- Field |
- Constr |
- Method


org.cloudbus.cloudsim.network.datacenter

## Class NetworkVm

- java.lang.Object
- - org.cloudbus.cloudsim.Vm
  - - org.cloudbus.cloudsim.network.datacenter.NetworkVm

- All Implemented Interfaces:
  :   java.lang.Comparable<java.lang.Object>

  ---

    

  ```
  public class NetworkVm
  extends Vm
  implements java.lang.Comparable<java.lang.Object>
  ```

  NetworkVm class extends Vm to support simulation of networked datacenters. It executes actions
  related to management of packets (send and receive).
  Please refer to following publication for more details:
  Saurabh Kumar Garg and Rajkumar Buyya, NetworkCloudSim: Modelling Parallel Applications in Cloud
  Simulations, Proceedings of the 4th IEEE/ACM International Conference on Utility and Cloud
  Computing (UCC 2011, IEEE CS Press, USA), Melbourne, Australia, December 5-7, 2011.

  Since:
  :   CloudSim Toolkit 3.0

- - ### Field Summary

    Fields

    | Modifier and Type | Field and Description |
    | --- | --- |
    | `java.util.ArrayList<NetworkCloudlet>` | `cloudletlist` |
    | `double` | `finishtime` |
    | `boolean` | `flagfree` |
    | `double` | `memory` |
    | `java.util.ArrayList<HostPacket>` | `recvPktlist` |
  - ### Constructor Summary

    Constructors

    Constructor and Description

    `NetworkVm(int id,
    int userId,
    double mips,
    int pesNumber,
    int ram,
    long bw,
    long size,
    java.lang.String vmm,
    CloudletScheduler cloudletScheduler)`
  - ### Method Summary

    Methods

    | Modifier and Type | Method and Description |
    | --- | --- |
    | `int` | `compareTo(java.lang.Object arg0)` |
    | `boolean` | `isFree()` |

    - ### Methods inherited from class org.cloudbus.cloudsim.Vm

      `addStateHistoryEntry, getBw, getCloudletScheduler, getCurrentAllocatedBw, getCurrentAllocatedMips, getCurrentAllocatedRam, getCurrentAllocatedSize, getCurrentRequestedBw, getCurrentRequestedMaxMips, getCurrentRequestedMips, getCurrentRequestedRam, getCurrentRequestedTotalMips, getHost, getId, getMips, getNumberOfPes, getRam, getSize, getStateHistory, getTotalUtilizationOfCpu, getTotalUtilizationOfCpuMips, getUid, getUid, getUserId, getVmm, isBeingInstantiated, isInMigration, setBeingInstantiated, setBw, setCloudletScheduler, setCurrentAllocatedBw, setCurrentAllocatedMips, setCurrentAllocatedRam, setCurrentAllocatedSize, setHost, setId, setInMigration, setMips, setNumberOfPes, setRam, setSize, setUid, setUserId, setVmm, updateVmProcessing`
    - ### Methods inherited from class java.lang.Object

      `clone, equals, finalize, getClass, hashCode, notify, notifyAll, toString, wait, wait, wait`

- - ### Field Detail


    - #### cloudletlist

      ```
      public java.util.ArrayList<NetworkCloudlet> cloudletlist
      ```


    - #### recvPktlist

      ```
      public java.util.ArrayList<HostPacket> recvPktlist
      ```


    - #### memory

      ```
      public double memory
      ```


    - #### flagfree

      ```
      public boolean flagfree
      ```


    - #### finishtime

      ```
      public double finishtime
      ```
  - ### Constructor Detail


    - #### NetworkVm

      ```
      public NetworkVm(int id,
               int userId,
               double mips,
               int pesNumber,
               int ram,
               long bw,
               long size,
               java.lang.String vmm,
               CloudletScheduler cloudletScheduler)
      ```
  - ### Method Detail


    - #### isFree

      ```
      public boolean isFree()
      ```


    - #### compareTo

      ```
      public int compareTo(java.lang.Object arg0)
      ```

      **Specified by:**
      :   `compareTo` in interface `java.lang.Comparable<java.lang.Object>`


- Overview
- Package
- Class
- Use
- Tree
- Deprecated
- Index
- Help

- Prev Class
- Next Class

- Frames
- No Frames

- All Classes

- Summary:
- Nested |
- Field |
- Constr |
- Method

- Detail:
- Field |
- Constr |
- Method
